# Intensive field phenotyping of maize (*Zea mays* L.) root crowns identifies phenes and phene integration associated with plant growth and nitrogen acquisition

**DOI:** 10.1093/jxb/erv241

**Published:** 2015-06-03

**Authors:** Larry M. York, Jonathan P. Lynch

**Affiliations:** ^1^Department of Plant Science, The Pennsylvania State University, University Park, PA 16802, USA; ^2^Ecology Graduate Program, The Pennsylvania State University, University Park, PA 16802, USA

**Keywords:** Capture, corn, interaction, root system architecture, RSA, soil, synergism, trait, uptake

## Abstract

Root architecture is an important regulator of nitrogen (N) acquisition. Existing methods to phenotype the root architecture of cereal crops are generally limited to seedlings or to the outer roots of mature root crowns. The functional integration of root phenes is poorly understood. In this study, intensive phenotyping of mature root crowns of maize was conducted to discover phenes and phene modules related to N acquisition. Twelve maize genotypes were grown under replete and deficient N regimes in the field in South Africa and eight in the USA. An image was captured for every whorl of nodal roots in each crown. Custom software was used to measure root phenes including nodal occupancy, angle, diameter, distance to branching, lateral branching, and lateral length. Variation existed for all root phenes within maize root crowns. Size-related phenes such as diameter and number were substantially influenced by nodal position, while angle, lateral density, and distance to branching were not. Greater distance to branching, the length from the shoot to the emergence of laterals, is proposed to be a novel phene state that minimizes placing roots in already explored soil. Root phenes from both older and younger whorls of nodal roots contributed to variation in shoot mass and N uptake. The additive integration of root phenes accounted for 70% of the variation observed in shoot mass in low N soil. These results demonstrate the utility of intensive phenotyping of mature root systems, as well as the importance of phene integration in soil resource acquisition.

## Highlight

Root phenes were phenotyped on all whorls of field-grown maize for the first time, and their integration could explain up to 70% of shoot mass variation in low nitrogen soils.

## Introduction

Global food security is a pre-eminent challenge of the 21st century (Funk and Brown, 2009), and food production must increase by at least 100% to meet the requirements of the 9.5 billion people predicted by 2050 (Royal Society of London, 2009; World Bank, 2014). The fact that ~1 billion people of the 7 billion now living are experiencing hunger (Godfray *et al.*, 2010) accentuates this pressing need. Farming more land is not a viable solution for this problem (Pretty, 2008), so land use efficiency must increase dramatically. Optimizing plant nutrient use efficiencies is one way to use land more productively (Lynch, 1998), especially because in much of the developing world, use of nitrogen (N) and phosphorus (P) fertilizers is negligible (FAO, 2012). In developed nations, much of the applied fertilizers are not taken up by plants and eventually pollute water and the atmosphere (Jenkinson, 2001). Maize is grown on 177 Mha, with aggregate yield exceeding all other grains (FAO, 2012), and is a mainstay of both subsistence and commercial agriculture. Reducing the fertilizer requirement of maize by developing genotypes with improved nutrient acquisition is an important goal for both subsistence and commercial agriculture (Lynch, 2007).

Root system architecture (RSA) has important effects on soil resource acquisition, plant interactions, and nutrient cycling in agricultural systems (Lynch, 1995, 2007; Hirel *et al.*, 2007; Zhang *et al.*, 2014) and natural systems (Mahall and Callaway, 1992; Comas and Eissenstat, 2009). The identification of root phenes (i.e. elemental units of the phenotype, *sensu* Serebrovsky, 1925; Lynch and Brown, 2012; see York *et al.*, 2013 for discussion), and understanding their utility for soil resource acquisition is an important step in phene-based or ideotype breeding critical for crop improvement (Kell, 2011; Lynch, 2014). The maize root system is comprised of an embryonic root system consisting of the primary root (radicle) and seminal roots emerging from the scutellar node, and successive whorls of nodal roots that emerge from the shoot (Hochholdinger, 2009). In the root system architectural taxonomy adopted by the International Society for Root Research, the primary root, seminal roots, and nodal roots of maize would be classified as the tap root, basal roots, and shoot-borne roots, respectively (Zobel and Waisel, 2010).

Many root architectural, anatomical, and morphological phenes and phene aggregates influence water and nutrient uptake, and root distribution in maize, including crown (below-ground nodal) root number (York *et al.*, 2013; Saengwilai *et al.*, 2014*b*), topsoil foraging (Zhu *et al.*, 2005), crown root angle (Trachsel *et al.*, 2013), lateral branching (Postma *et al.*, 2014; Zhan and Lynch, 2015), root cortical aerenchyma (Zhu *et al.*, 2010; Saengwilai *et al.*, 2014*a*), living cortical area (Jaramillo *et al.*, 2013), and cortical cell size and file number (Chimungu *et al.*, 2014*a* , *b*). These same phenes may influence competition and facilitation among plants (Rubio *et al.*, 2003; Zhang *et al.*, 2014). Understanding how these root phenes interact with one another to give rise to a functionally integrated phenotype in different environments is a complex challenge; however, analysis based on root phene integration may also reveal phene modules consisting of several positively interacting phenes that may be co-selected (York *et al.*, 2013). Even though many of these root architectural phenes have been associated with functional utility in the field, little is known about variation of root phenes among the nodes of the maize root system and whether this variation could have functional importance for soil resource acquisition.

Variation of root phenes within the maize root system is difficult to study because the outer whorls of the maize root system are the youngest roots and occlude the older roots in the interior. When root phene differences among nodes are studied, only one line may be reported (e.g. Picard *et al.*, 1985), or else an incomplete measurement of only a few nodes is conducted for many lines (e.g Guingo *et al.*, 1998). One approach to increase throughput of field phenotyping is the use of digital images of excavated root crowns combined with automatic image analysis (Grift *et al.*, 2011; Bucksch *et al.*, 2014); however, the use of mature root systems prevents measurements of the occluded part of the root system, and image analysis platforms are currently unable to count individual nodal roots. The use of mesocosms containing clear gels allows detailed architectural analysis over time; however, growth is limited to young plants, and such media may introduce artefacts due to their dissimilarity to field soil (Iyer-Pascuzzi *et al.*, 2010). X-ray computed tomography is another promising approach to visualize roots over time in soil; however, pot size is currently a limitation (Mooney *et al.*, 2011). Measurements of nodal root growth angles in the field have generally been limited to the outer brace (above-ground nodal) and crown roots (Trachsel *et al.*, 2011). Screening of maize seminal root systems has been conducted extensively on germination paper in the lab (Zhu *et al.*, 2006; Hund *et al.*, 2009), but has not been confirmed by field experiments and does not provide insight about the nodal root system. Phenotyping methods exist along a continuum between intensive, with more measurements, and extensive, being conducted on many varieties (Fiorani and Schurr, 2013), with trade-offs in speed and costs due to labour and instruments. The trajectory of the field of plant phenotyping tends towards maximizing the extent and intensity of phenotyping.

While progress is being made in relating root phenes to soil resource acquisition in maize, these phenes are commonly measured on only a single whorl or else aggregated across all whorls. In order to test the hypotheses that root architectural phenes are influenced by the node on which they manifest, that variation in root architectural phenes within the root system can be as great or greater than variation observed among genotypes, and that phenes of all whorls affect N uptake and plant growth, the root crowns of several maize lines were intensively phenotyped under two N levels in the field.

## Materials and methods

Similar field experiments were conducted in Pennsylvania in the USA in 2012 and in Limpopo province in South Africa in 2014 (details below). Because the overall results were similar between the two sites and to provide more detail in the main text, all results from South Africa and the most pertinent data from the USA are presented in the main text. Comprehensive data from the USA are provided as supplementary data available at *JXB* online.

### Plant material

Twelve recombinant inbred lines (RILs) were used from the intermated B73×Mo17 (IBM) population (Sharopova *et al.*, 2002) including line numbers: 5, 13, 15, 63, 85, 121, 167, 172, 195, 222, 317, and 351. These 12 RILs were originally selected to have contrasting nodal root growth angles.

### Experimental site

The field site was near Alma, Limpopo province, South Africa (24°33′00.12S, 28°07′25.84E, 1235 m asl) with the soil being a Clovelly loamy sand (Typic Ustipsamment). The average temperature between planting and sampling was 22 °C, total rainfall was 340mm, and average relative humidity was 70%.

### Treatment installation

The experiment included four blocks within a 20 ha centre pivot irrigated field. Each field was split in half randomly to create split-plots in which to create a high nitrogen (HN) and a low nitrogen (LN) soil environment. Entire fields received P and potassium as determined by soil tests. A planter passed through all fields leaving behind non-planted rows in which to plant manually. Within the HN and LN split-plots, IBM lines were randomly assigned to plots. Each plot contained five rows that were 4 m long with a row distance of 76cm. Seeds were hand-planted on 23 November 2013 into rows marked by the planter using stakes and ropes marked to accommodate a density of 80 000 plants ha^–1^. At planting, 23kg N ha^–1^ was applied to the entire field through centre pivot fertigation, and an additional 23kg N ha^–1^ was supplied as granular urea in the HN split-plots. HN split-plots received 46kg N ha^–1^ as granular urea approximately every 3 weeks to reach a total of 184kg N ha^–1^. Additional water was supplied as needed with centre pivot irrigation. Micronutrients were supplied at a rate of 3kg ha^–1^ 60 d after planting.

### Experimental sampling and harvest

Shoots and root crowns were sampled from two blocks each day between 4 and 5 February 2014 (72–73 days after planting). Three plants were chosen from each plot based on their average size and being surrounded by other plants. The entire plants were excavated and processed in the field from each plot using the shovelomics method (Trachsel *et al.*, 2011), with the shovel inserted 30cm from the base of the plants. The three root crowns were soaked in water then rinsed with a water hose and nozzle until most soil was removed. One root crown was selected for subsequent architectural measurements based on its apparent average size and uniformity (i.e. least diseased, least nematode damage, not containing a large stone, etc.).

Shoots from the three plants were combined and dried at 60 °C for 5 d before being weighed. The entire leaf from above the ear was dried and ground in a Wiley mill with a 40-mesh sieve (Thomas Scientific, Waltham, MA, USA) and a subsample analysed for N content with an elemental analyser (PerkinElmer 2400 Series II, Swedesboro, NJ, USA).

### Root crown imaging and architectural measurement

**Fig. 1.**
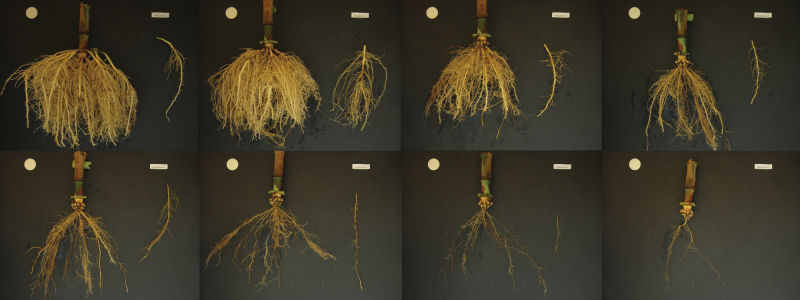
A mature root crown of maize is dissected by excising whorls of nodal roots from the outside to expose the next layer and imaging with a digital camera. In this series of images, whorls are excised from outer to inner, from left to right, and top to bottom. Top left depicts the outermost brace roots at node position 7, while crown roots at node position 1 are on the bottom, second from right, and the seminal root system, not measured, is at bottom right. Root phenes were measured for every nodal whorl.

The original shovelomics method (Trachsel *et al.*, 2011) was modified to accelerate field processing while permitting more intensive measurements. Root crowns were kept in large plastic bins submerged in water inside a 5 °C cold room until they were imaged within 1 week. Root crowns were imaged using digital cameras attached to frames with camera mounts such that the camera was facing down from a height of 50cm. Three identical cameras (PowerShot A1200, Canon, Melville, NY, USA) operated by three researchers were used to process samples quickly. Root crowns were placed under the camera on a matte black background. A 3cm white plastic disk was included as a scale in every image, along with a printed sample label. Camera zoom and focus were kept locked for the duration of the imaging. An image was taken of every whorl of nodal roots (Fig. 1) by removing all roots in a node sequentially. A representative nodal root (average diameter, not diseased) was excised from the side of the root crown not facing the camera for each whorl and placed to the side of the root crown such that both root crown and representative nodal root were in frame of the image. Imaging all whorls of a maize root crown required ~10min on average, depending on the number of whorls and the number of roots in each whorl.

Image analysis was conducted in RSAJ which is a project for the ObjectJ plugin (Vischer and Nastase, 2014) for ImageJ (Schneider *et al.*, 2012). RSAJ and its manual are available at http://plantscience.psu.edu/roots/methods/computer/RSAJ. RSAJ prompts the user to take sequential measurements from the images (Supplementary Fig. S1 at *JXB* online), which are briefly described (see the RSAJ manual for elaboration). Nodal root growth angle (NRGA) from the horizontal was derived trigonometrically from the stem width (SW), maximum root crown width, and the height between stem width and root crown width (see manual). The number of nodal roots (nodal occupancy, NO) counted for a whorl was multiplied by 2 in order to account for the occluded half of the root system, based on previous observations of symmetry in maize root crowns. The diameter of the representative nodal root (nodal root diameter, NRD) was measured, along with the distance from the basal cut to where lateral roots emerge (distance to branching, DTB). In order to calculate lateral root branching density (LRBD), the number of lateral roots was counted along a measured length on the representative nodal root. Finally, the lengths of three representative lateral roots (lateral root length, LRL) were measured and averaged for analysis. However, because lateral roots may break during excavation and washing, the apparent lateral root length measured here may underestimate the total length. Distances in pixels were converted to centimetres or millimetres by using the pixel width of the circular 3cm scale. See Table 1 for a list and of all measured root phenes and their abbreviations. Images were renamed prior to analysis with all experimental information and node position. Node position was labelled in the order of development, with position 1 being the oldest (or coleoptilar) node (Hund *et al.*, 2011). Image analysis required ~1min for each image.

**Table 1.** Phenes measured within maize root crowns at each node position are listed with their abbreviations, the source of the measurement (from the root crown or the representative nodal root), and a brief description

Phene	Abbreviation	Source	Description
Stem width	SW	Crown	Stem width at a node
Nodal occupancy	NO	Crown	The number of nodal roots in a node
Noda root growth angle	NRGA	Crown	The angle from horizontal in a node
Nodal root diameter	NRD	Root	The thickness at the base of a nodal root from a node
Distance to branching	DTB	Root	The length from the base of a nodal root to first lateral
Lateral root branching density	LRBD	Root	The number of lateral roots from a nodal root in 1 cm
Lateral root length	LRL	Root	The average of three lateral roots from a nodal root

### Statistical analysis

All statistics were conducted and data graphics were created with R version 3.0.2 (R Core Team, 2013). Analysis of variance (ANOVA) was conducted with mixed effects modelling using the R function *nlme* with N level nested within block as the random effect and N level, genotype, and node position as the fixed effects (Table 2). In order to quantify the proportion of variation in root phenes contributed by the node position and genotype, the effect size η^2^ was calculated from ANOVA sums of squares (Table 3). Linear regressions were conducted separately for HN and LN between each root phene in each whorl individually against shoot mass, percentage N in the leaves, and total leaf N content with data from all genotypes. Phenes with regression *P*-values <0.1 were combined for multiple regression and then stepwise regression was used to find the most parsimonious model predicting shoot mass, percentage N, and total N from root phenes using the *step* function in R. The data for averages of the four replicates for N level, genotype, and node combinations (*n*=165, incomplete rows omitted) were centred and scaled using the *scale* function, and then the *prcomp* function was used to conduct multivariate principal component analysis (PCA) in R. Root crown phene aggregates were derived from the raw data, including total nodal root number and the number of nodes for each root crown, and analysed by *t*-test for an N effect. Graphs were created in R with the *ggplot2* package (Wickham 2009).

**Table 2.** ANOVA table of maize root phenes giving the F-value and significance for all factors and factor interactions

	SW	NRGA	NO	NRD	DTB	LRBD	LRL
Nlevel	77.87**	0.32NS	12.54*	26.05*	16.44*	1.6NS	7.2NS
Geno	13.4**	5.32**	3.16**	3.73**	8.21**	12.23**	6.72**
Node	3263.4**	156.6**	822.33**	3349.2**	113.01**	107.45**	228.6**
Nlevel:Geno	4.27**	1.83*	0.63NS	2.15*	1.52NS	0.91NS	2.63**
Nlevel:Node	67.45**	3.23NS	13.45**	16.04**	26.13**	1.78NS	2.52NS
Geno:Node	10.85**	3.94**	1.81 *	5.64**	2.99**	2.78**	3.3 **
Nlevel:Geno:Node	3.74**	0.97NS	0.29NS	1.17NS	1.24NS	1.51NS	0.68NS

Phene abbreviations are as in Table 1.

**P ≤0.01; *0.01<*P*≤0.05; NS, *P*>0.05, not significant.

**Table 3.** Effect size as η^2^ demonstrates the amount of variation (%) explained by each factor, interaction, and the residuals in the ANOVA

	SW	NRGA	NO	NRD	DTB	LRBD	LRL
Nlevel	3.25	0.15	1.90	1.01	2.54	0.21	1.89
Geno	3.14	6.53	2.28	0.95	10.08	14.85	7.34
Node	71.26	17.31	53.60	80.01	12.54	11.70	22.62
Nlevel:Geno	1.16	2.43	0.47	0.58	1.88	1.20	3.00
Nlevel:Node	1.45	0.36	0.87	0.37	2.88	0.22	0.25
Geno:Node	2.66	4.79	1.34	1.54	3.67	3.47	3.60
Nlevel:Geno:Node	0.92	1.15	0.20	0.31	1.52	1.98	0.79
Residuals	16.16	67.28	39.34	15.24	64.89	66.37	60.51

Phene abbreviations are as in Table 1.

## Results

### Influence of node position, genotype, and N level on phene values

Stem width, averaged across N level and genotype, generally increased from 0.41cm in the first node to ~1.5cm in the last nodes (Table 2; Supplementary Fig. S2 at *JXB* online; *P*<0.01). Among genotypes, stem width, averaged across node positions and N levels, ranged between 0.83cm and 1.15cm (*P*<0.01). Stem width decreased from 1.06cm under HN to 0.89cm under LN (*P*<0.01). All interactions were significant. The node position explained 71.3% of the variation in stem width, while genotype explained 3.1% (Table 3).

**Fig. 2.**
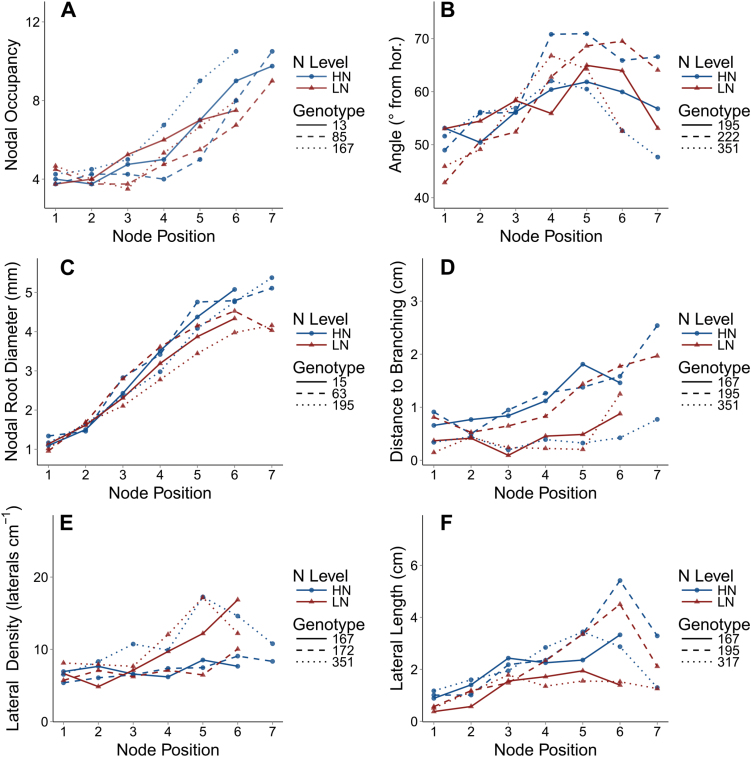
Six root phenes were measured for every whorl in high and low nitrogen soil, and data for three contrasting genotypes are shown here. Node position 1 is the oldest whorl. (A–F) Plots of nodal occupancy, nodal root growth angle, nodal root diameter, distance to branching, lateral root density, and lateral root length, respectively. Points are the average of the four replicates. Within each panel, data from high nitrogen (HN) are depicted with filled circles and in low nitrogen (LN) with filled triangles, and different genotypes are represented by line types. Data for all phenes and all genotypes with standard errors are included in the Supplementary Figs S2–S8 at *JXB* online.

Nodal occupancy (NO) increased with the node position, from an average of four roots in the first whorl to an average of 11 roots in the last whorl (Table 2; Fig. 2A; Supplementary Fig. S3 at *JXB* online; *P*<0.01). Average nodal occupancy among genotypes ranged between 5.3 and 6.8 roots (*P*<0.01). Average nodal occupancy decreased from 6.2 in HN to 5.5 in LN (*P*<0.038). The rate of change and total number of roots differed among genotypes, where some produced four roots on the first 3–4 whorls before increasing, while others began producing more roots earlier (node×genotype significant interaction). The node position explained 53.6% of the variation in NO, while genotype explained 2.28% (Table 3).

Nodal root growth angle (NRGA) from the horizontal was affected by node position and ranged between 51.7 ° and 54.8 ° in the first three whorls and between 60.2 ° and 65.4 ° in the last six whorls, averaged across genotype and N level (Table 2; Fig. 2B; Supplementary Fig. S4 at *JXB* online; *P*<0.01). Among genotypes, averaged across node position and N level, NRGA ranged between 54.2 ° and 62.0 º (*P*<0.01). The effect of N level, overall, was not significant. Generally, the angle increased (became steeper) from the first to the fourth node position, and from the fourth position some genotypes showed an increased angle, some remained the same, and others showed a decreased angle (significant interactions). The node position explained 17.3% of the variation in NRGA, while genotype explained 6.5% (Table 3).

Nodal root diameter (NRD) increased with node position from 1.0mm in the first whorl to ~4.5mm in the last whorls (Table 2; Fig. 2C; Supplementary Fig. S5 at *JXB* online; *P*<0.01). Among genotypes, averaged across node position and N level, NRD ranged from 2.7mm to 3.2mm (*P*<0.01). NRD decreased from 3.0mm in HN to 2.7mm in LN (*P*=0.015). Generally, the increase with node position was linear, though in some cases diameter levelled off with the transition from crown to brace roots around the sixth node position. The node position explained 80% of the variation in NRD, while genotype explained 1% (Table 3).

The distance to branching (DTB), on average, increased from 0.56cm in the first node to 2.0cm in the last node (Table 2; Fig. 2D; Supplementary Fig. S6; at *JXB* online; *P*<0.01). However, not all genotypes exhibited this increase with node position (significant genotype×node interaction). DTB among genotypes, averaged across node positions and N levels, ranged from 0.40cm to 1.2cm (*P*<0.01). DTB decreased from 0.95cm in HN to 0.70cm in LN (*P*=0.027). The node position explained 12.5% of the variation in DTB, while genotype explained 10% (Table 3).

Lateral root branching density (LRBD), on average, increased with node position from 7.3 lateral roots cm^–1^ in the first node to 13.0 lateral roots cm^–1^ in the last nodes (Table 2; Fig. 2E; Supplementary Fig. S7 at *JXB* online; *P*<0.01), although this increase does not occur for all genotypes (significant node×genotype interaction). Among genotypes, averaged across node position and N level, LRBD ranged from 6.9 to 10.9 lateral roots cm^–1^ (*P*<0.01). N level had no significant effect on LRBD. The node position explained 11.7% of the variation in LRBD, while genotype explained 14.9% (Table 3).

Lateral root length (LRL), on average, increased with the node position from 0.64cm in the first node to 3cm in the last node (Table 2; Fig. 2F; Supplementary Fig. S8 at *JXB* online; *P*<0.01). Among genotypes, averaged across node position, LRL ranged between 1.25cm and 2.44cm (*P*<0.01). N level had no significant effect on LRL. The node position explained 22.6% of the variation in LRL, while genotype explained 7.3% (Table 3).

Results regarding the relationship of node position to measured root phenes from the USA are presented as Supplementary Figs S12–S16 at *JXB* online.

### Influence of nitrogen on root crown phene aggregates, nodal root number, and number of nodes

**Fig. 3.**
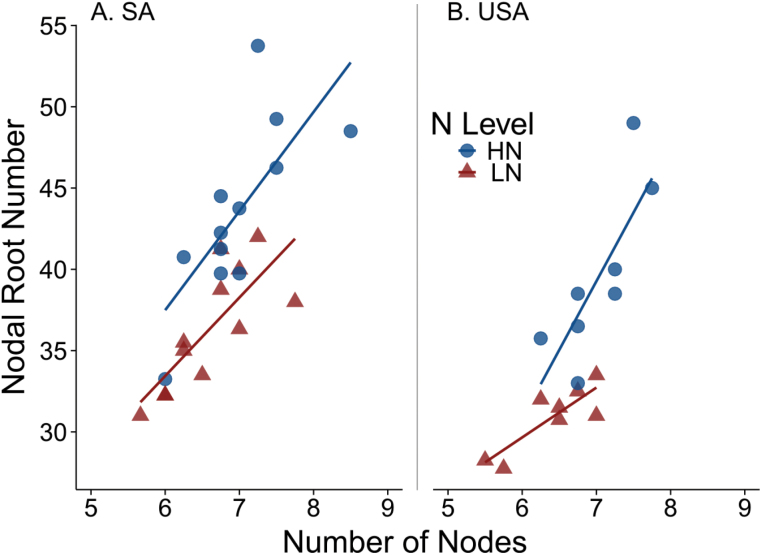
Scatter plots and linear regressions are shown for the relationship of the total number of nodes in a root crown with the total number of nodal roots in a root crown in both South Africa (SA, A) and the USA (B). Points are the mean of four replicates for each genotype. Data from high nitrogen (HN) are depicted with filled circles and in low nitrogen (LN) with filled triangles. Solid lines indicate the linear model of best fit for HN (*y*=6.09*x*+0.92; *P*=0.0066) and LN (*y*=4.82*x*+4.49; *P*=0.00266) in SA. Solid lines indicate the linear model of best fit for HN (*y*=8.44*x*–19.83; *P*=0.0177) and LN (*y*=3.07*x*+11.26; *P*=0.0082) in the USA. (This figure is available in colour at *JXB* online.)

Nodal root number (NRN), the combined number of nodal roots in an entire root crown, ranged between 31 and 53.75 among genotypes and N levels in South Africa (Fig. 3A). Nodal root number decreased 16% from 44 under HN to 36 under LN (*P*<0.01) in South Africa. The total number of nodes within a root crown varied between 5.7 and 8.5 among genotypes and N levels in South Africa (Fig. 3A). The number of nodes averaged across genotypes decreased 6% from seven nodes under HN to 6.6 nodes under LN (*P* =0.022). The linear models predicting nodal root number from the number of nodes in both HN (*y*=6.09*x*+0.92; *P*<0.01) and LN (*y*=4.82*x*+4.49; *P*<0.01) were significant in South Africa. In the USA, the linear models predicting nodal root number from number of nodes in both HN (*y*=8.44*x*–19.83; *P*=0.0177) and LN (*y*=3.07*x*+11.26: *P*=0.0082) were also significant (Fig. 3B). Correlations between total number of nodal roots and the occupancy of each whorl generally showed relationships between adjacent whorls in South Africa. In LN, the occupancies of whorls 2 and 6 were most correlated with NRN (Supplemetnary Fig. S9 at *JXB* online). In HN, the occupancies of whorls 3, 5, and 7 were most correlated with NRN (Supplementary Fig. S10).

### Principal component analysis

**Fig. 4.**
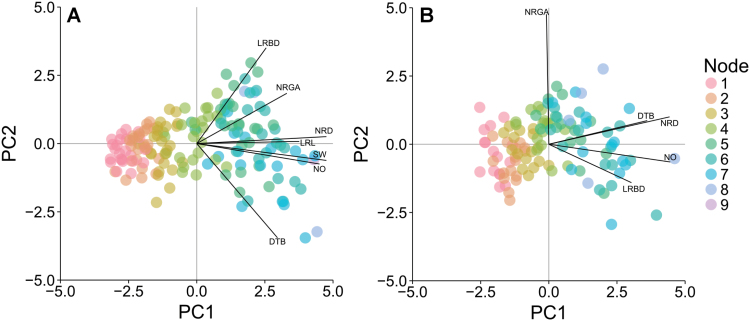
Principal component analysis of root architectural phenes conducted on data averaged across the four replicates for each nitrogen level, maize genotype, and node position combination for SA (A) and the USA (B). Points represent the scores of principal components 1 and 2 (PC1 and PC2) for each nitrogen level, maize genotype, and node position combination. Labelled lines demonstrate the correlation of phene values to principal component scores (maximum correlation, 0.951, SA; 0.952, USA). Abbreviations are as given in Table 1.

PCA of the average phene values for N level, genotype, and node position combinations in South Africa revealed two principal components, PC1 and PC2, that explained 60% and 16% of the total variation, respectively (Fig. 4A). In the USA, PC1 and PC2 explained 49% and 21% of the total variation (Fig. 4B). In both experimental sites, PC1 was greatly influenced by nodal root diameter and nodal occupancy, but in South Africa lateral root length and stem width also contributed. In South Africa, PC2 was primarily influenced by distance to branching, lateral root branching density, and nodal root growth angle. PC2 was dependent on nodal root growth angle only in the USA. At both sites, the scores of PC1 were heavily dependent on node position, where younger whorls had greater PC1 scores. Correlational analysis supports the structure of these components, where nodal root diameter, lateral root length, stem width, and nodal occupancy were strongly correlated with each other and with the node position in South Africa (Supplementary Fig. S11 at *JXB* online).

### Relationships among root phenes, shoot mass, and N content

**Fig. 5.**
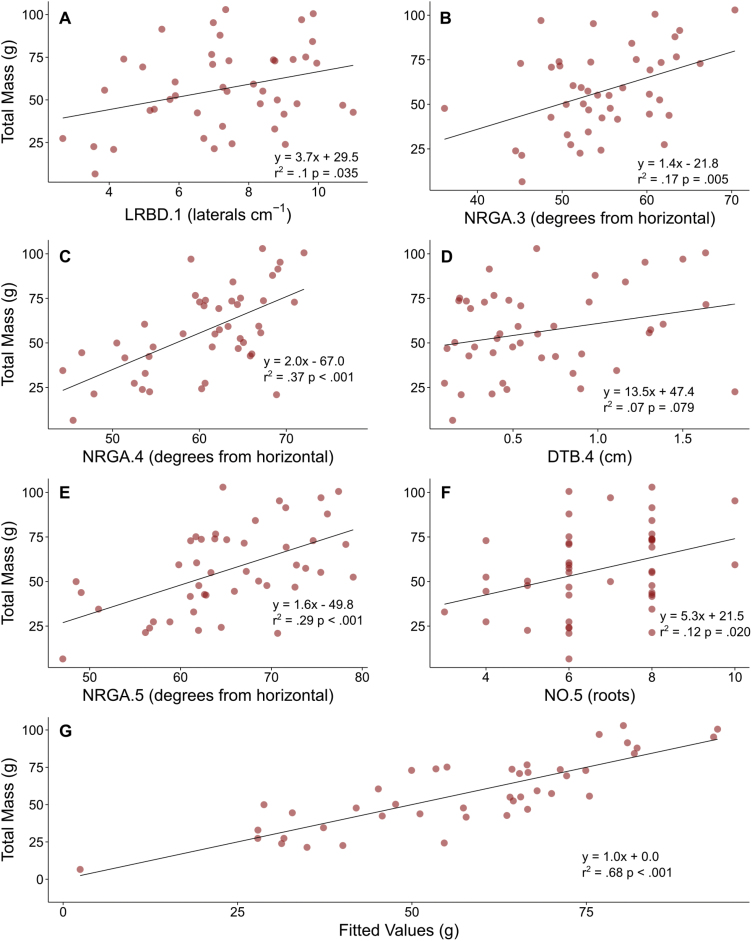
Multiple panels show the effect of the most significant and explanatory phenes from all whorls on total shoot mass in low nitrogen plots after stepwise multiple linear regression. (A–F) The relationship of the following phenes to total shoot mass: LRBD.1, NRGA,3, NRGA.4, DTB.4, NRGA.5, and NO.5. Abbreviations are as given in Table 1, and the appended number identifies the whorl in which the phene was measured. (G) Fitted values are calculated from the linear combinations of the above phenes using the coefficients determined by multiple linear regression. (This figure is available in colour at *JXB* online.)

**Fig. 6.**
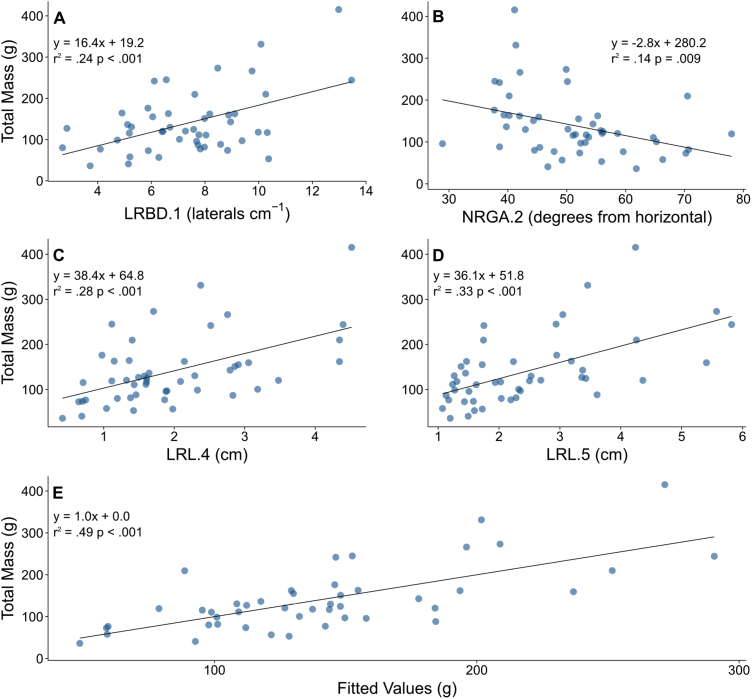
Multiple panels show the effect of the most significant and explanatory phenes from all whorls on total shoot mass in high nitrogen plots after stepwise multiple linear regression. (A–D). The relationship of the following phenes to total shoot mass: LRBD.1, NRGA,2, LRL.4, LRL.5, NRGA.5, and NO.5. Abbreviations are as given in Table 1, and the appended number identifies the whorl in which the phene was measured. (E) Fitted values are calculated from the linear combinations of the above phenes using the coefficients determined by multiple linear regression. (This figure is available in colour at *JXB* online.)

In LN, linear regression of all root phenes from all whorls against shoot mass identified 24 phenes with significant relationships. Stepwise regression of the most significant root phenes of different whorls in LN, and not including stem widths, revealed a model containing +LRBD.1, +NRGA.3, +NRGA.4, +DTB.4, +NRGA.5, and +NO.5 (numerical suffix denotes the node position, + and – indicating positive and negative relationships, respectively) as the most parsimonious model which accounted for 69% of the variation in shoot mass (Fig. 5). In HN, linear regression of all root phenes from all whorls against shoot mass identified 22 phenes with regression *P*-values <0.1. Stepwise regression of the most significant root phenes of different whorls in HN, and not including stem widths, revealed a model containing +LRD.1, –NRGA.2, +LRL.4, and +LRL.5 as the most parsimonious model which accounted for 49% of the variation in shoot mass (Fig. 6). In LN, a multiple regression model including the nodal occupancies of all whorls explained 34% of shoot mass variation, while a regression model with total nodal root number explained 22%. In HN, neither the multiple regression model of all whorl occupancies nor the regression model with NRN were significant. Percentage reduction in shoot mass was calculated for every genotype and block combination, then all root phenes were regressed, which identified 12 root phenes with regression *P*-values <0.1. Stepwise regression of these root phenes identified –NRGA.4, –NRD.5, and –NRN as the most parsimonious model, explaining 33% of the variation in percentage reduction in shoot mass (*P*<0.01).

Linear regression of percentage N in leaves from plants grown in LN soil as a response to all root phenes in all whorls identified 14 phenes with regression *P*-values <0.1. Stepwise regression of the most significant root phenes of different whorls, not including stem widths, revealed a model containing –LRL.2, +NRD.3, and –LRBD.6 as the most parsimonious model, which accounted for 36% of the variation in leaf percentage N (*P*<0.01). In HN, no root phene had a significant effect on percentage leaf N. The results of regressions of total leaf N content were largely driven by the effect of leaf mass and are similar to the shoot mass results above, so are not included. Log transformations of the data did not substantially influence the results and most relationships appear linear.

## Discussion

This study presents a novel analysis of the variation of several root architectural phenes both within and among maize root crowns for 12 genotypes and how integration of root architectural phenes affects performance in low- and high-N soils. The most sensitive phenes to node position were size-related phenes such as stem width, nodal occupancy, and nodal root diameter, and these phenes tended to have greater variation within root crowns than among genotypes. However, some phenes had considerable variation both within root crowns and among genotypes, especially distance to branching and lateral root length. Nodal root growth angle had the least variation, both within root crowns and among genotypes. Nodal root number and the number of nodes varied greatly among genotypes and N levels, and both were decreased by N level. There was a significant positive correlation between the number of nodes and the nodal root number. PCA demonstrated that most variation is explained by size-related phenes and the first principal component could discriminate node position. In general, relationships among root phenes of different whorls and among root phenes and shoot properties such as mass and percentage N demonstrate the importance of measuring the root phenes of all whorls. More phenes correlated with shoot mass of plants grown in LN soil than in HN soil, especially NRGA of several whorls, and these phenes were different from the phenes that correlated with shoot mass of plants grown in HN soil. The differing relationships of phenes with biomass in LN and HN suggest that the relationships are not allometric (i.e. are not inherently related to plant size; Niklas, 2004). Stepwise multiple linear regression suggests that the additive integration of several phenes, each with small effects, can explain a large amount of the variation observed in shoot mass, in this case almost 70%.

Most studies measuring maize root architecture in the field focus on the outer whorls of brace and crown roots (Trachsel *et al.*, 2011, 2013; Grift *et al.*, 2011; Bucksch *et al.*, 2014), though these whorls arguably contribute the least to soil resource acquisition because they emerge late and into zones of soil already explored by other roots. This study demonstrates that a more detailed analysis of the root system benefits physiological studies quantifying the functional utility of specific root phenes. Phenes measured at the node level may be more elemental and useful for genetic studies as well, supported by the lack of constant relationships among phenes among nodes in different genotypes. Recently, intensive phenotyping of lateral root phenes of several orders was accomplished using semi-automated methods in one maize hybrid (Wu and Guo, 2014). Dissection of the root crown also allows the counting of nodes, which could be an important phene because of its relationship to total nodal root number. However, this method only allows basic understanding of the temporal dynamics of node emergence which was shown to be important in simulations (York, 2014). Weekly sampling conducting similar RSA measurements may be important for determining variation in the emergence times of nodes. Earlier work has documented the increase in nodal occupancy with node position (Picard *et al.*, 1985; Hoppe *et al.*, 1986; Stamp and Kiel, 1992), but differences among genotypes were not reported nor was the number of nodes described as an independent root phene of maize previously.

Maize root phenotypes with fewer crown roots were recently demonstrated to increase N capture, shoot mass, and yield in low N soils (Saengwilai *et al.*, 2014*b*), confirming earlier simulation results (York *et al.*, 2013). In the current study, a positive relationship between NRN and shoot mass existed for plants grown in LN soil, but not for plants grown in HN soil, which suggests the relationship in LN was not necessarily allometric because variation existed for NRN and shoot mass in HN but they were not correlated. In this case, more nodal roots may be associated with greater vigour. Bayuelo-Jiménez *et al.* (2011) also found a positive correlation between nodal root number and shoot mass in 50 maize accessions in high and low P soils. In LN soil, NRN was dependent mostly on the occupancies of the second and sixth whorls, so intensive phenotyping of this phene aggregate by decomposition into more elemental phenes such as nodal occupancies for every whorl could benefit breeding programmes. A multiple regression model of shoot mass including the linear combinations of the nodal occupancies of every whorl was more explanatory than NRN alone, which again demonstrates the importance of more intensive phenotyping. Screening for fewer root nodes in a maize breeding programme could be a simple and fast method to initiate a programme selecting for fewer nodal roots, providing an example of traditional selection followed by precision selection discussed by Cobb *et al.* 2013.

In some genotypes used in this study, nodal root growth angles were steeper in younger whorls, which agrees with earlier reports (Feldman, 1994). However, in other genotypes, NRGA was almost the same in all whorls or else was more shallow in younger whorls. Studies of a single maize genotype are often extrapolated to the species level, yet here and elsewhere substantial variation for almost any measured root phene was demonstrated so genotype to species level extrapolations are generally not warranted. Measuring NRGA on only outer whorls is not a reliable way to predict the overall pattern of growth angles among whorls. In LN soil, steeper angles of older and younger whorls were associated with greater shoot mass, while in HN shallow roots in whorl 2 were slightly associated with greater shoot mass. In this experiment, N was only applied in LN plots once then leached deeper, while N was applied several times in HN plots so constituted a generally shallow resource. The ‘steep, cheap, and deep’ (SCD) ideotype for optimal water and N foraging by maize root systems hypothesized that steeper angles would allow greater acquisition of leaching N (Lynch, 2013). Conversely, shallow-angled roots will acquire more shallow resources such as N applied several times or P. Plasticity of the nodal root growth angle has previously been observed in maize, with shallow-angled genotypes in HN becoming up to 18 º steeper in LN (Trachsel *et al.*, 2013); however, consistent NRGA plasticity was not observed in the current study. In common bean, plasticity of the basal root growth angle was determined to be under independent genetic control, with some genotypes becoming more steep and some more shallow in response to low P (Bonser *et al.*, 1996). Steep-angled nodal roots were hypothesized to benefit maize plants grown with deficits of deep resources such as leaching nitrate or water during terminal drought (Lynch, 2013), while shallow roots of maize are known to increase P uptake (Zhu *et al.*, 2005) and possibly uptake of other shallow resources.

Increased lateral root branching density of the first whorl was associated with increased shoot biomass in both the LN and HN soils of this study, and ranged between ~4 and 10 lateral roots cm^–1^. Recently, simulation studies concluded that between 6 and 10 lateral roots cm^–1^ optimize nitrate acquisition depending on the nitrate levels, with substantial declines in plant dry weight beyond 10 lateral roots cm^–1^ (Postma *et al.*, 2014). The same simulation study concluded that at least 10 lateral roots cm^–1^ optimize phosphate acquisition, with no decline in plant dry weight if lateral root branching density is increased further. The maximum of ~10 lateral roots cm^–1^ in the first whorl found in this study could suggest co-optimization of both N and P acquisition during early growth. Early plant vigour is an important characteristic of maize ideotypes (Mock and Pearce, 1975), and in this study the stem width at the first node (a proxy for early growth) was positively correlated with total shoot mass (LN, *y*=146*x*–3.5, *r* ^2^=0.18, *P*=0.004; HN, *y*=441*x*–39.9, *r* ^2^=0.24, *P*<0.001).

In the HN soils, but not in LN soils, increased lateral root length of the fourth and fifth whorls correlated strongly with plant shoot mass. Long laterals may increase the volume of soil exploited for nitrate without creating substantially more competition among roots of the same plant, and long, sparse laterals are a component of the SCD ideotype (Lynch, 2013). This hypothesis was supported by a simulation study in maize (Postma *et al.*, 2014) which showed that the optimal lateral root branching density was greater for P acquisition than for N acquisition, and reduced branching density led to longer laterals which benefitted nitrate capture. These simulation results were supported by a study showing that maize genotypes with fewer but longer lateral roots had greater N acquisition from low N soil in the field and in greenhouse mesocosms (Zhan and Lynch, 2015). Longer laterals deserve further consideration as a possible breeding target.

Distance to branching generally increases along with the length of laterals on nodal roots, which agrees with recent work demonstrating a 50% increase in the distance to branching over the past 100 years in commercially successful maize (York *et al.*, 2015). Possibly, greater distance to branching frees metabolic resources to invest in longer laterals and minimizes the placement of lateral roots in soil already explored by nodal roots from older nodes. Greater distance to branching is a novel phene state that complements the existing ‘steep, cheap, and deep’ ideotype (Lynch, 2013).

### Root phene integration

The integration of root phenes will determine how multiple phenes interact through their influence on soil resource foraging and plant metabolic status (York *et al.*, 2013). Studying the integration of root phenes in the maize root crown required the intensive phenotyping used in this study in order to understand the root crown as a whole constructed of more elemental constituents. Applying a phene-based paradigm necessitates a continual re-evaluation of what properties are elemental and developmentally unique (Lynch and Brown, 2012), such as by measuring the properties of individual whorls rather than crown-level aggregates. Ambiguity may exist as to what extent a phene is elemental, but the attempt to clarify the study of the plant phenome will be aided by a more precise conceptual definition of a phene and, correspondingly, more precise terminology.

The additive multiple linear regression models used in this study show that considering multiple phenes together offers predictive power for understanding plant growth. Phene interactions are rarely considered in root biology, or are purposely minimized by comparing isophenic lines in order to evaluate the function of individual phenes (Zhu *et al.*, 2005; Chimungu *et al.*, 2014*a*; Saengwilai *et al.*, 2014*b*; Zhan and Lynch, 2015). However, notable exceptions include integration of root hair phenes in *Arabidopsis thaliana* simulations (Ma *et al.*, 2001), integration of root cortical aerenchyma and lateral root branching in maize simulations (Postma and Lynch, 2011), and a field experiment demonstrating synergism between shallow basal root growth angle and many, long root hairs in common bean (*Phaseolus vulgaris*) (Miguel *et al.*, 2015). Interestingly, an experiment of a single maize variety grown in aeroponics found a plastic response to low N where the number of crown roots was reduced, while the length of laterals increased (Gaudin *et al.*, 2011), which may demonstrate physiological integration of maize that generates a more N-efficient root system phenotype through developmental modifications that balance carbon costs of different root classes.

### Intensive phenotyping platforms

Most root phenotyping platforms either screen seedlings on germination paper in the lab, or screen mature root crowns in the field without measuring the occluded, older nodal roots. Though time consuming, if intensive phenotyping was performed on hundreds of maize lines, genome-wide association studies (GWAS; reviewed by Cobb *et al.*, 2013) could be conducted that might identify the genetic basis for phene states that either vary independently or vary dependently among whorls. Studying the development and genetics of roots among whorls could contribute as much insight into the regulation of root growth as studying the differences among genotypes considering the greater variation of root phenes observed among whorls than among genotypes. Intensive phenotyping may complement extensive phenotyping by targeting the most relevant diversity panels or subsets of lines based upon the results of extensive phenotyping.

Wu and Guo (2014) presented novel semi-automated methods to excavate maize root crowns, wash the crowns, measure root phenes, and create visualizations. The extension and improvement of these methods could provide a means to sample large populations. Many imaging methods from gels, to X-rays, to field-based imaging use aggregate measurements or mathematical descriptors of the entire root crown and so obscure the underlying variation within maize root crowns (Iyer-Pascuzzi *et al.*, 2010; Grift *et al.*, 2011; Mooney *et al.*, 2011; Bucksch *et al.*, 2014). However, these same methods could provide the basis of allowing intensive phenotyping to be used extensively on large diversity panels as long as the phenes of different nodes can be measured independently. Recently, a 6 df (degrees of freedom) digitizer was used to reconstruct a three-dimensional model of two maize hybrids grown in the field and to extract information about the curvature of several whorls of nodal roots in maize, which might be another promising method for field phenotyping (Wu *et al.*, 2014). Another recent automated image analysis method used maize crowns that were split in half in order to reveal the interior structure and provided novel insights, yet all whorls were aggregated for measurements (Colombi *et al.*, 2015). Despite the proliferation of research on phenotyping maize root phenes and studying root phene utility for soil resource acquisition in recent years, the field is still developing and requires advances in both the intensity and extent of phenotyping. Because of the inherent temporal dynamics of spatial availability of different soil resources, variation within the maize root crown as revealed by intensive phenotyping may reflect adaptations to allow maize genotypes to have different spatiotemporal foraging properties. The SCD ideotype hypothesized that earlier forming axial roots should have many laterals while later forming axial roots should have fewer but longer lateral roots (Lynch, 2013). This study suggests that the decoupling of relationships of phene states among whorls may be possible, but to what extent and benefit is not known.

Near-isophenic plants have similar phenotypic backgrounds and allow the opportunity to compare specific contrasts of phenes without the confounding influences of variation in many phenes (Chimungu *et al.*, 2014*a* , *b*; Saengwilai *et al.*, 2014*a*, *b*; Zhan and Lynch, 2015). Biparental RIL populations are useful for these phenotypic contrasts, but also have limited phenotypic diversity which limits their usefulness for studying the utility of root phenes. Structured diversity panels with greater phenotypic variation provide another opportunity for studying root phenes, though more advanced statistics may be needed to account for the influence of variation in several phenes (Mezmouk *et al.*, 2011).

Functional–structural plant modelling will continue to advance the study of root phenes (Dunbabin *et al.*, 2013). Simulations allow more detailed studies of root system functioning than possible in greenhouse and field studies. Simulation models permit the study of phenotypes that do not exist in nature, and many different climates, soils, and soil resource levels can be simulated. Field experiments are costly in terms of land use, labour, and supplies, so simulation modelling allows much larger experimental designs. Simulation modelling informs empirical work, and at the same time empirical work provides the data and insights for new simulations, so modelling and empirical research are synergistic (Wullschleger *et al.*, 1994).

## Conclusion

Intensive phenotyping of all whorls within the maize root crown revealed that while many size-related root phenes generally increased with younger node positions, other phenes, such as NRGA, DTB, LRBD, and LRL, can have different patterns depending on the genotype. Statistical models of the additive integration of several root phenes accounted for a large proportion of the variance in shoot mass, up to 70% in LN soil. Intensive phenotyping of both root architectural and anatomical phenes is rarely attempted, but architectural and anatomical variation in root phenes was described in many lines from different *Zea* species (Burton *et al.*, 2013), and a recent report studied a single maize cultivar and reported several architectural and anatomical phenes for several classes of roots (Gao *et al.*, 2015). Recently, phenotyping architectural and anatomical phenes in maize hybrids representing varieties grown over the past 100 years in the USA demonstrated evolution of the maize root system towards phene states efficient for N acquisition (York *et al.*, 2015). Phenotyping that is both intensive and extensive, coupled to GWAS, could be a powerful technique to accelerate the understanding of how the integration of root phenes among whorls affects soil resource acquisition in maize and other crops with potential impacts for global food insecurity.

## Supplementary data

Supplementary data are available at *JXB* online.

Figure S1. Screenshot of root measurements using RSAJ.

Figures S2–S8. Detailed plots of variation among nodes, N levels, and genotypes for root phenes in South Africa.

Figures S9–S11. Correlations among phenes in high and low N in South Africa.

Figures S12–S16. Detailed plots of variation among nodes, N levels, and genotypes for root phenes in the USA.

RSAJ Manual

Supplementary Data
