# Supplementary material for: Intensive field phenotyping of maize (Zea mays L.) root crowns identifies phenes and phene integration associated with plant growth and nitrogen acquisition
Source: J Exp Bot. 2015 Jun 3;66(18):5493–505. doi: 10.1093/jxb/erv241 (PMC4585417; doi:10.1093/jxb/erv241)
Supplement: Supplementary Data [file supp_66_18_5493__index.html]

Intensive field phenotyping of maize (Zea mays L.) root crowns identifies phenes and phene integration associated with plant growth and nitrogen acquisition — Intensive field phenotyping of maize (Zea mays L.) root crowns identifies phenes and phene integration associated with plant growth and nitrogen acquisition — Supplementary Data 

# Intensive field phenotyping of maize (*Zea mays* L.) root crowns identifies phenes and phene integration associated with plant growth and nitrogen acquisition

## Supplementary Data

Data files

**Files in this Data Supplement:**

- Supplementary Data - Supplementary Data
